# The ontology of biological sequences

**DOI:** 10.1186/1471-2105-10-377

**Published:** 2009-11-18

**Authors:** Robert Hoehndorf, Janet Kelso, Heinrich Herre

**Affiliations:** 1 Research Group Ontologies in Medicine, Institute for Medical Informatics, Statistics and Epidemiology, University of Leipzig, Haertelstrasse 16-18, 04107 Leipzig, Germany; 2 Department of Evolutionary Genetics, Max Planck Institute for Evolutionary Anthropology, Deutscher Platz 6, 04103 Leipzig, Germany; 3 Department of Computer Science, University of Leipzig, Johannesgasse 26, 04103 Leipzig, Germany

## Abstract

**Background:**

Biological sequences play a major role in molecular and computational biology. They are studied as information-bearing entities that make up DNA, RNA or proteins. The Sequence Ontology, which is part of the OBO Foundry, contains descriptions and definitions of sequences and their properties. Yet the most basic question about sequences remains unanswered: what kind of entity is a biological sequence? An answer to this question benefits formal ontologies that use the notion of biological sequences and analyses in computational biology alike.

**Results:**

We provide both an ontological analysis of biological sequences and a formal representation that can be used in knowledge-based applications and other ontologies. We distinguish three distinct kinds of entities that can be referred to as "biological sequence": chains of molecules, syntactic representations such as those in biological databases, and the abstract information-bearing entities. For use in knowledge-based applications and inclusion in biomedical ontologies, we implemented the developed axiom system for use in automated theorem proving.

**Conclusion:**

Axioms are necessary to achieve the main goal of ontologies: to formally specify the meaning of terms used within a domain. The axiom system for the ontology of biological sequences is the first elaborate axiom system for an OBO Foundry ontology and can serve as starting point for the development of more formal ontologies and ultimately of knowledge-based applications.

## Background

Biological sequences play a major role in genetics and bioinformatics research. They are important in the description of DNA, RNA and proteins, and are among the basic entities studied in molecular and computational biology. In the realm of biological ontologies, the Sequence Ontology (SO) [1] was developed to describe sequences and their features semantically. Although many formal definitions are available for the SO categories, several categories remain defined using natural language.

Formal ontologies are intended to formally specify a conceptualization of a domain [2], and therefore provide the foundation for data and information integration and exchange. Definitions alone are insufficient to achieve this goal. Axioms are required to provide meaning for primitive, undefined categories and relations. To provide an ontological analysis of "biological sequence" and to formalize the basic categories used in the SO, several ontological questions about sequences must be answered, among them: what kind of entity is a biological sequence? How does it relate to space and time? What are the entities that necessarily have to exist for a sequence to exist? What are the properties of biological sequences? What relations are applicable to sequences? How do sequences relate to other kinds of entities, in particular to molecules, organisms or processes (of selection and mutation)?

Here we provide both an ontological analysis of *biological sequence *and an axiom system for the SO's top-level categories. We use first- and second-order logics for this purpose. The axiom system is intended to serve as a foundation for the SO, and as a means to achieve interoperability between the SO and other domain ontologies through the provision of an explicit formalization of the basic categories and relations used in the context of sequences. For the construction of the axiom system, we employed the axiomatic method [3]. The axiom system is freely available for download from our project page [4].

## Method

Our investigation of the ontology of sequences is based on the axiomatic method and on principles of ontological analysis [3,5,2]. We consider a formal ontology to be a specification of a conceptualization, i.e., a system of categories representing a particular view on the world [6,2]. A formal ontology uses a vocabulary whose terms denote concepts and relations which refer to things in reality.

One method that is used to specify the meaning of a term is an *explicit definition*. An explicit definition for a relation or category *P *provides a sentence *ϕ *in which *P *does not occur, such that every occurrence of *P *can be replaced with *ϕ*. For example, to define *RedCircle *as an entity which is both red and a circle, we could write the definition:(1)

Because this is a definition, whenever we use *RedCircle *in any statement, we can replace it with the right side of the definition, i.e., *red*(*x*) ⋀ *circle*(*x*). This leaves a statement in which *RedCircle *does not occur, but *red *and *circle *instead.

When explaining the meanings of a set of terms through explicit definitions, other terms must be used to define the terms in the set, and in turn the meaning of these terms must be specified (without creating a circular definition). Therefore, specifying the meanings of terms solely through explicit definitions will either lead to an infinite regress or leave several terms unspecified. In the latter case, the meaning of all terms for which a definition is provided depends on the meaning of the terms without definition.

We call the terms that are not explicitely defined *primitive terms*. The meaning of all terms in the ontology depends on the meaning of these primitive terms: because non-primitive terms are introduced through explicit definitions, every sentence involving a non-primitive term can be replaced with a sentence containing only primitive terms. For example, the defined category *RedCircle *can be replaced by the right side of equation 1 in every statement in which it occurs. Subsequently, *red *and *circle *can be replaced by their *definiens *if they are defined. Therefore, every statement can be transformed in a statement that consists only of primitive terms.

The problem remains how the meaning of the primitive terms can be described formally. For this purpose, we construct sentences containing only primitive terms. These sentences can be understood as descriptions of formal interrelations between the primitive terms. Some of these sentences are chosen as axioms: they are accepted as being true within the domain under consideration. Such axioms provide restrictions on the interpretation of the primitive terms, and therefore on the terms defined using these primitive terms. For a formal theory, and therefore for a formal ontology, the axioms are the central component, because only they can give significant meaning to terms used in the theory. Furthermore, the axioms are chosen in a way such that further true statements can be deduced from the axioms.

It is important to note that definitions do not add significant meaning to a term as long as the terms in the definition remain unspecified. Without axioms, only trival statements hold for the primitive terms, i.e., the logically valid statements. Formally, non-trivial meaning can only be established through appropriate axioms for the primitive terms.

## Results

The theory of biological symbols and sequences that we propose here is intended to be compatible with the Sequence Ontology (SO) [1]. The SO uses two basic categories in the characterization of sequences, *region *and *junction*. Both can have attributes, i.e., properties, and sub-categories. For example, a sequence may be a *gene *or a *base*, a junction an *insertion site*, and a sequence attribute *enzymatic*.

Sequences are linear entities and can come in two facets. Sequences can either have a start and an end point or form circles. There are sequence atoms which are usually denoted by single letters. These atoms have no proper sequence parts.

The use of the term *sequence *in the SO permits different interpretations. Here, we introduce an important distinction that is currently neglected in the SO. The SO contains as their only basic category a sequence region, and employs an extensional mereological system on it. However, we will show that it is important to distinguish between "sequence" as abstract, information-bearing entity, the physical manifestation as a *molecule *and the syntactic representation of a "sequence". To illustrate the difference between an abstract sequence and its physical manifestations (tokens), consider all constituents of the sequences *ACAC *and *CAAC*. The first sequence has as parts - or sequence motifs - the sequences *ACAC*, *ACA*, *CAC*, *AC*, *CA*, *A *and *C*. The sequence *CAAC *has as parts the sequences *CAAC*, *CAA*, *AAC*, *CA*, *AA*, *AC*, *A *and *C*. It is remarkable that, although both sequences apparently have the same *length*, use the same primitive symbols (only *A *and *C*), and each primitive symbol occurs exactly twice in each sequence, *ACAC *has seven sequences as part, while *CAAC *has eight. This is due to the fact that, in one sense of "sequence", there is *only one AC*, which occurs in *ACAC twice*. On the other hand, each molecular *token *of *ACAC *and of *CAAC *will have at least ten parts: two adenine molecules, two cytosine molecules, three molecules consisting of two nucleotides each, two molecules consisting of three nucleotides each, and the whole molecule (because **part-of **is reflexive).

The problem arises because *sequence *has different meanings which result in different properties. When a sequence is understood as a *pattern*, then the parts of a sequence are patterns too. In this case, in the sequence *ACAC *or a longer repeat of *AC*, there are only two subpatterns of length two: *AC *and *CA*. Both *AC *and *CA *are patterns which are a part of the longer pattern *ACAC *or a repeat of *AC*. When a sequence is understood as a molecule, the parts of a sequence are molecules (or residues), and any molecular sequence of a length *n *will have *n *- 1 molecular sequences of length two as part.

### Overview, primitive categories and basic definitions

The theory we propose here assumes that *Abstract sequence *(*ASeq*), *Syntactic sequence *(*SSeq*), *Molecular sequence *(*MSeq*) and *Junction *(*Jun*) are primitive categories. In particular, they are not defined, but characterized axiomatically according to the axiomatic method.

Instances of the *Molecular sequence *category are molecules or residues, the elements in chains of nucleotides or amino acids that are designated by single letters in their representation. A single nucleotide or a single amino acid is an instance of *Molecular sequence*. A nucleotide residue which is part of a DNA molecule is an instance of *Molecular sequence*, and so is an amino acid residue which is part of a protein. We consider these entities - single nucleotide or amino acid residues - as atomic instances of *Molecular sequence*; they have no proper parts which are themselves instances of *Molecular sequence*. Non-atomic instances of *Molecular sequence *are primary structures of polynucleotides or proteins, i.e., chains of monomeric subunits. *Molecular sequence *does not include chemical molecules that are not nucleotides, amino acids or chains thereof. In particular, it is not equivalent to the category of all chemical molecules, but a proper sub-category.

An instance of *Abstract sequence *is an abstract entity. It is independent of space and time: either the instances of *Abstract sequence *are not located in space and time, or they are located everywhere and at all times. Intuitively, an *Abstract sequence *represents an equivalence class of sequence tokens or representations. Therefore, an *Abstract sequence ***A **unites that which all A-tokens have in common. There is only one *Abstract sequence *instance **A**. Abstract sequences can have abstract sequences as parts: the abstract sequence **ACAC **has the abstract sequences **ACA**, **CAC**, **AC**, **CA**, **A **and **C **as proper parts. Both **A **and **C **occur *twice *in each token of **ACAC**. There is only one abstract sequence **A **and **C **(which represent the equivalence classes of the two **A**- and **C**-tokens in **ACAC**). Therefore, the abstract sequence **ACAC **has the abstract sequence **A **and the abstract sequence **C **as part *only once*.

We use a third category *Syntactic sequence *in our axiom system. Instances of *Syntactic sequence *are sequence representations. They are representations in biological databases, textual representations in the form of strings or graph-based representations. They represent the arrangement of the molecules in the molecular sequences, and stand for an abstract sequence. Instances of *Syntactic sequence *are usually material entities, such as patterns of ink or configurations of magnetic fields on electromagnetic storage media. An instance of *Syntactic sequence *can be atomic when it does not have proper parts, such as for the syntactic sequence *A*.

Atomic instances are delimited by instances of *Junction*, i.e., boundaries between two atomic parts of a sequence representation. Instances of *Junction *represent chemical bonds or binding sites on the level of molecular sequences. The category *Junction *corresponds to the category SO:0000699 (*Junction*) of the SO, which is "a boundary between regions".

**Figure 1:**
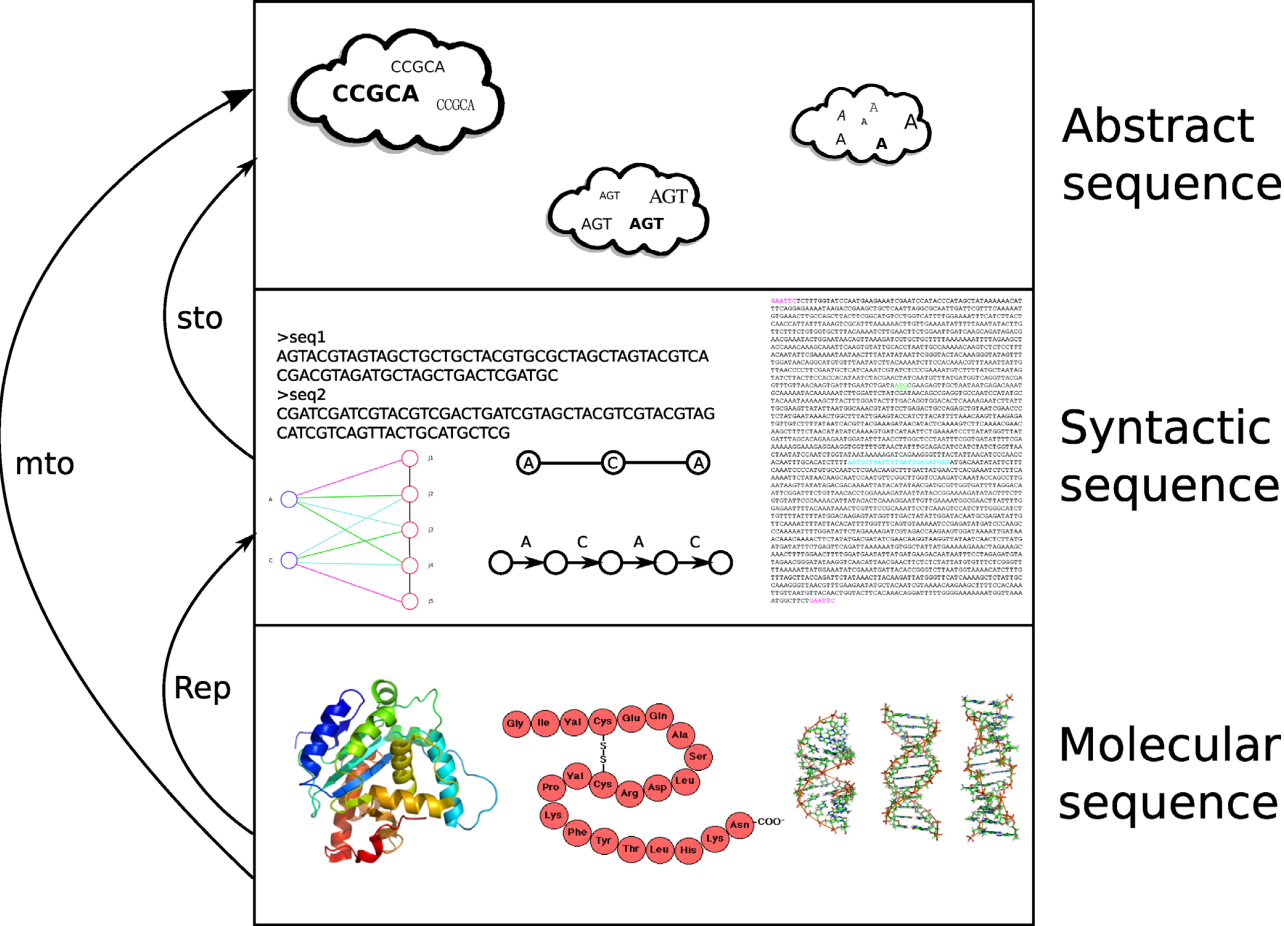
**Different layers of sequences**. We illustrate the layers in the ontology of sequences. At the bottom level, sequences can refer to chains of molecules. These chains correspond to the primary structures of DNA and RNA molecules as well as proteins. The middle level illustrates sequences as representations of molecules. These can be in different formats such as the FASTA file format, plain text, graph-based representations or similar. Sequence representations exhibit a syntactic structure that resembles the structures of molecule chains. However, not every instance of *Syntactic sequence *represents a chain of molecules; sequence representations can represent no, one or many molecules. The upper level shows abstract sequences, symbolized as equivalence classes of sequence representations.

A schematic overview of the layers of our axiom system and their interrelations is illustrated in figure 1. Table 1 lists the relations and predicates we use in the axiom system.

**Table 1:** List of relations and predicates.

Symbol	Long name of predicate	Remarks
*MSeq*(*x*)	molecular sequence	
*SSeq*(*x*)	syntactic sequence	
*ASeq*(*x*)	abstract sequence	
*Jun*(*x*)	junction	
*PBS*(*x*)	primitive biological symbol	*x *is a syntactic sequence (*SSeq*).
*DMSeq*(*x*)	directed molecular sequence	*x *is a molecular sequence (*MSeq*).
*DSSeq*(*x*)	directed syntactic sequence	*x *is a syntactic sequence (*SSeq*).
*mPO*(*x, y*)	molecular part of	*x *and *y *are molecular sequences (*MSeq*).
*sPO*(*x, y*)	syntactic part of	*x *and *y *are syntactic sequences (*SSeq*).
*aPO*(*x, y*)	abstract part of	*x *and *y *are abstract sequences (*ASeq*).
*mPPO*(*x, y*)	molecular proper part of	*x *and *y *are molecular sequences (*MSeq*).
*sPPO*(*x, y*)	syntactic proper part of	*x *and *y *are syntactic sequences (*SSeq*).
*aPPO*(*x, y*)	abstract proper part of	*x *and *y *are abstract sequences (*ASeq*).
*moverlap*(*x, y*)	molecular overlap	*x *and *y *are molecular sequences (*MSeq*).
*soverlap*(*x, y*)	syntactic overlap	*x *and *y *are syntactic sequences (*SSeq*).
*aoverlap*(*x, y*)	abstract overlap	*x *and *y *are abstract sequences (*ASeq*).
*mdisjoint*(*x, y*)	molecular disjointness	*x *and *y *are molecular sequences (*MSeq*).
*sdisjoint*(*x, y*)	syntactic disjointness	*x *and *y *are syntactic sequences (*SSeq*).
*adisjoint*(*x, y*)	abstract disjointness	*x *and *y *are abstract sequences (*ASeq*).
*sto*(*x, y*)	syntactic token of	*x *is a syntactic (*SSeq*), *y *an abstract sequence (*ASeq*).
*mto*(*x, y*)	molecular token of	*x *is a molecular (*MSeq*), *y *an abstract sequence (*ASeq*).
*Rep*(*x, y*)	representation	*x *is a syntactic (*SSeq*), *y *a molecular sequence (*MSeq*).
*between*(*j, p*_1_, *p*_2_, *s*)	between	*j *is a junction (*Jun*), *p*_1 _and *p*_2 _are primitive symbols (*PBS*) and *s *is a syntactic sequence (*SSeq*). *j *is a junction between *p*_1 _and *p*_2 _in the syntactic sequence *s*.
*end*(*j, p, s*)	ends	*j *is a junction (*Jun*), *p *a primitive symbol (*PBS*) and *s *is a syntactic sequence (*SSeq*). The junction *j *ends the syntactic sequence *s *and is adjacent to the primitive symbol *p *(which is the first or last symbol of *s*).
*first*(*j, p, s*)	first	*j *is a junction (*Jun*), *p *a primitive symbol (*PBS*) and *s *a syntactic sequence (*SSeq*).
*last*(*j, p, s*)	last	*j *is a junction (*Jun*), *p *a primitive symbol (*PBS*) and *s *a syntactic sequence (*SSeq*).
*in*(*j, s*)	in	*j *is a junction (*Jun*) and *s *a syntactic sequence (*SSeq*).
*s*_1 _≡ *s*_2_	equivalence	*s*_1 _and *s*_2 _are directed syntactic sequences (*DSSeq*).
*conn*(*j*_1_, *j*_2_)	connection	*j*_1 _and *j*_2 _are junctions (*Jun*).

The table shows the list of predicates used in the axiom system. Unary predicates represent categories, all other predicates represent relations. In this table, we included relations that are used in the implementation but are not further discussed. For example, the relations *adisjoint *and *mdisjoint *are included in the axiom system and are defined similar to *sdisjoint *(see formula 13).

The central relations in our axiom system are part-whole relations. We provide axioms for three different kinds of part-whole relations, one for each level of representation. The relation **mPO **(molecular-part-of) relates instances of *Molecular sequence*, **sPO **(syntactic-part-of) relates instances of *Syntactic sequence *and **aPO **(abstract-part-of) relates instances of *Abstract sequence*. Additionally, the *representation *relation (**Rep**) relates instances of *Molecular sequence *to instances of *Syntactic sequence*. The relation **sto **(syntactic-token-of) relates instances of *Syntactic sequence *to instances of *Abstract sequence*, while the relation **mto **(molecular-token-of) relates instances of *Molecular sequence *to instances of *Abstract sequence*.

We use several relations that are more technical in nature to specify molecules and their representations. For this purpose, we use the **binds **relation that represents a chemical bond between two molecules, and the relations **conn**, **in**, **between **and **end **to describe instances of *Syntactic sequence *and how they relate to *Junction*. The relation **conn **holds between two connected junctions in a syntactic sequence. The relation ≡ is a relation between two syntactic sequences that are tokens of the same abstract sequence. In our ontological analysis and the resulting axiom system, we make no commitment to a particular ontological system. The ontology of sequences presented here can stand on its own, and axioms are presented for all relations used in the theory. However, the foundation in a top-level ontology can benefit the interoperability between the presented ontology and other domain-specific ontologies, because the top-level ontology can provide a common interface for multiple domain ontologies. Therefore, we discuss options for a foundation in top-level ontologies after presenting our axiom system.

### Basic axioms

The first part consists of axioms that restrict the arguments of some of the relations. The remaining relations take defined categories as arguments and are introduced later. Additionally, an axiom requiring all sequences to have only molecules as tokens is introduced.(2)

The following set of axioms requires that *Junction*, *Molecular sequence*, *Syntactic sequence *and *Abstract sequence *are disjoint categories, i.e., not two of these categories have an instance in common.(6)

### Sequence mereology

The relation **sPO **is a parthood relation that holds for syntactic sequences when one sequence contains the other as a sequence part. Based on this relation we first define **sPPO **(syntactic proper sequence part of) and the category of primitive biological symbols (*PBS*) as well as the **soverlap **and **sdisjoint **relations.

A syntactic sequence *x *is a proper part of the syntactic sequence *y *if *x *is a syntactic part of *y *but not identical to *y*. A syntactic sequence is primitive (*PBS*) if it has no proper parts. Two sequences *x *and *y *overlap if they have a part in common (i.e., if there is a part *z *of *x *which is also a part of *y*), and they are disjoint if they do not overlap.(10)

The relation **sPO **satisfies reflexivity, transitivity and antisymmetry, and therefore forms a partial order.(14)

The relation **sPO **also satisfies the strong supplementation principle, leading to an extensional mereology for sequences [7,8]:(17)

Syntactic sequences consist entirely of atoms (primitive biological symbols) with respect to the relation **sPO**. The following two axioms require that all sequences have primitive biological symbols as part, and that they are constituted of only primitive biological symbols:(18)

As a result of these axioms, the relation **sPO **specifies an atomic extensional mereology for the instances of *Syntactic sequence*.

### Sequences, junctions and connectivity

The second part of axioms pertaining to sequences relates the symbols to junctions. Intuitively, junctions are borders between two adjacent primitive symbols and delimiters at the beginning and end of a syntactic sequence.

First, we restrict the arguments for the **between**, **end **and **conn **relations, and introduce the relation **in **through an explicit definition. A junction is **in **a sequence *s *if it is either a junction between two primitive biological symbols within *s*, or it ends the sequence *s *and is adjacent to exactly one primitive biological symbol (eqn. 23). The relations **between **and **end **represent these two cases. The relation **conn **asserts that two junctions follow each other within a sequence.(20)

The relations **between **and **end **are introduced for technical reasons and are not intended for direct use. They are used to specify a syntactic sequence token as a chain of primitive biological symbols separated by junctions. An assertion *between*(*j*, *p*_1_, *p*_2_, *s*) is read as "*j *is a junction between the primitive biological symbols *p*_1 _and *p*_2 _in the sequence *s*". The relation **end **serves a similar purpose. Both relations are used to define the relation **in**, and we will make use of the **in **relation in the following axioms.

The following set of axioms pertains to the **conn **relation of connectedness between junctions. The relation is used to represent the order of the sequence through an order of junctions.(24)

The axioms presented so far are first-order axioms, and they do not suffice to state that syntactic sequences must be connected. For this purpose, a second-order axiom is required. Equation 28 is an axiom in monadic second-order logic and states that the set of all junctions in a sequence, *P *= {*x*|*in*(*x*, *s*)}, is closed under the relation **conn**. In the axiom 28, *P *is the set of all junctions in some sequence *s *(*P*(*x*) ↔ *in*(*x*, *s*)). The second part of axiom 28 states that every non-empty subset *Q *of *P *(∃*aQ*(*a*) ⋀ ∀*x*(*Q*(*x*) → *P*(*x*)) which contains with every junction *u *also any junction *v *that is connected to *u *(*Q*(*u*) ⋀ *conn*(*u*, *v*) → *Q*(*v*)) is a superset of *P *(*P*(*x*) → *Q*(*x*)). Because *Q *is by construction both a non-empty subset and a superset of *P*, *P *and *Q *are equal. *Q *is closed against the relation **conn **and *P *is the set of junctions which are **in **a sequence. Therefore, axiom 28 states that the set of all junctions in a sequence is closed under the relation **conn**.(28)

Several more axioms that relate sequence representations to junctions can be found in the implementation of our axiom system. Similarily, a set of axioms that pertains to instances of the *Molecular sequence *category, including a mereological system and the relation to sequence representations, can be found in the full axiom system which is available from the project website.

### Directed and abstract sequences

In the axioms presented so far, instances of *Syntactic sequence *have no directionality. For many applications, it is useful to make sequence representations directional, i.e., determine a beginning and end. Such a definition is trivial for linear sequences. Linear sequences have exactly two junctions which **end **the sequence. When these are distinguished in a **first **and **last **junction, i.e., the two **end **junctions are distinguished, a directionality is immediately given, from **first **to **last**. For circular sequences, two arbitrary connected junctions are chosen as **first **and **last**. We use the predicate *DSSeq *in our axiom system to refer to directed sequences. Furthermore, we use *DMSeq *to refer to directed chains of molecules, which are formally constructed similarily to directed sequences.

Abstract sequences are abstracted from the sequence representations, and therefore indirectly from the tokens. Primitive biological symbols represent one abstract sequence directly. This can be considered as labelling the sequence representation with single letters representing individual tokens. Then, abstract sequences correspond to classes of sequence representations that are labelled with the same sequence of letters, i.e., whose primitive biological symbols each represent the same abstract sequence.

In the current version of the axiom system, we use the predicates *ASeq*(*x*), *Rep*(*x*, *y*) and ≡ (*s*_1_, *s*_2_). *ASeq*(*x*) means that *x *is an instance of *Abstract sequence*, *sto*(*x*, *y*) that the directed sequence *x *is a syntactic token of the abstract sequence *y*, *mto*(*x*, *y*) that the molecular sequence *x *is a molecular token of the abstract sequence *y*, and ≡ (*s*_1_, *s*_2_) that the directed sequences *s*_1 _and *s*_2 _are equivalent (i.e., are tokens of the same abstract sequence).

First, we restrict the arguments of the token-of relations and the equivalence relation between syntactic sequences.(29)

We will use the infix notation *s*_1 _≡ *s*_2 _instead of ≡ (*s*_1_, *s*_2_).

The following axioms ensure that directed sequences are syntactic tokens of one and only one abstract sequence. Therefore, the token-of relations are functional. We use the counting quantifier ∃(=1, *y*) to represent that there is one and only one *y *satisfying the conditions in the formula.(32)

Abstract sequences are dependent on their tokens: for every abstract sequences, there is at least one syntactic or molecular sequences that is the token of the abstract sequence.(34)

In the current state of the axiom system, we use a complex axiom to capture the equivalence between two directed sequences. Intuitively, two sequences are equivalent if and only if they are either primitive biological symbols that are tokens of identical abstract sequences or they *start *with equivalent primitive biological symbols and their proper parts that contain everything except these equivalent symbols are equivalent. This is a recursive definition which takes the form of an axiom in first order logics and is expressed in formula 35.(35)

Axiom 35 is not an explicit definition, because the relation ≡ appears on both sides of the formula. Instead, it represents a recursive definition in which the right side of axiom 35 contains only proper parts of the sequences that appear on the left side.

Axiom 35 can be read as follows: Two syntactic sequences *s*_1 _and *s*_2 _are equivalent (*s*_1 _≡ *s*_2_) if and only if they start with the primitive symbols *p*_1 _and *p*_2 _(*first*(*j*_1_, *p*_1_, *s*_1_) ⋀ *first*(*j*_2_, *p*_2_, *s*_2_)) and both *s*_1 _and *s*_2 _are primitive and tokens of the same abstract sequence (*PBS*(*s*_1_) ⋀ *PBS*(*s*_2_) ⋀ ∀*x*, *y*(*sto*(*s*_1_, *x*) and *sto*(*s*_2_, *y*) → *x *= *y*)) or they start with equivalent primitive symbols (*p*_1 _≡ *p*_2_) and those proper parts of *s*_1 _and *s*_2 _which contain everything of *s*_1 _and *s*_2 _except for the symbols *p*_1 _and *p*_2 _are equivalent.

With this axiom and based on **sto**, we can also characterize equivalence between directed sequences as a relation that holds if and only if the syntactic sequences are tokens of the same abstract sequence:(36)

Based on the representation relation **sto **and its axioms, we can define the relation **aPO**, which is a part-of relation for abstract sequences. An abstract sequence *x *is the abstract part of the abstract sequence *y*, if and only if there is a token *a *of the abstract sequence *y*, and *a *has a part (via **sPO **or **mPO**) that is the token of the abstract sequence *x*.(37)

As a corollary from this definition and the axioms pertaining to the **sto **relation and the equivalence of sequences, the **aPO **relation for abstract sequences groups tokens based on equivalence classes. In particular, our motivating example of the parts of the sequences *ACAC *and *CAAC *can be solved with the notion of abstract sequences and the **aPO **relation.

### Ontological foundation

A question that is not answered with these axioms is how sequences and junctions relate to categories commonly found in a top-level ontology. We believe these axioms to be compatible with most major top-level ontologies, in particular the Basic Formal Ontology (BFO) [9], the Descriptive Ontology for Linguistic and Cognitive Engineering (DOLCE) [10] and the General Formal Ontology (GFO) [11]. However, the foundation in these ontologies varies substantially.

In the BFO, molecular sequences should be considered as a subcategory of *Material entity*. Since syntactic sequences are also material entities, i.e., ink on paper, they can be represented as material entities as well. Junctions are specifically dependent continuants which depend on the syntactic sequences. A category *C *is specifically dependent on a category *D *if for every instance *c *of *C*, an instance *d *of *D *must exist, and *d *remains the same continuant throughout the life of *c*. Abstract sequences should be considered subcategories of *Generically dependent continuant*. A category *A *is generically dependent on the category *B *if and only if for every instance of *A*, some instance of *B *must exist. In the framework of the BFO, abstract sequences are generically dependent on either molecular or syntactic sequences. Because abstract sequences are generically dependent continuants, the dependency relations must be carefully examined for each sequence: many syntactic sequences considered in biology represent no molecular sequences, partially due to limitations in sequencing technology.

In the DOLCE, the category *Abstract *is a sub-category of *Particular*. The main characteristic of abstract entities is that they do not have spatial nor temporal qualities, and they are not qualities themselves.

Abstract sequences have this property, and can be embedded in DOLCE with the following axiom:(38)

Both syntactic and molecular sequences are sub-categories of *Endurant *in DOLCE, while junctions are qualities of syntactic sequences. The main difference between the foundation of our ontology of sequences in DOLCE and BFO is that, in DOLCE, abstract sequences are entities in their own right, independent of our creation of representations and independent of molecular manifestations of these sequences, while they are existentially dependent on their tokens in the BFO.

Integration of our theory in the GFO can be similar to the scenario described in the DOLCE, considering abstract sequences as a sub-category of GFO's *Abstract individual *category. However, the GFO also provides the category *Symbol structure*, of which abstract sequences can be a sub-category. Symbol structures are higher-order categories in the GFO. Higher-order categories are ontological categories that have categories as instances. In this case, the relations **sto **and **mto **are sub-relation of GFO's **token-of **relation, which is a sub-relation of the instantiation relation. The relation **sto **would relate one kind of tokens of abstract sequences, while the relation **mto **relates the other kind of tokens to abstract sequences. In the GFO, abstract sequences are entities in their own right, either abstract individuals or sub-categories of *Symbol structure*.

### Example: the sequences *ACAC *and *CAAC*

As motivating example for our investigation, we have used the sequences *ACAC *and *CAAC *and claimed that there are at least two views on these: one in which they each have ten parts, and one where they have different numbers of parts. We can now make this observation precise by distinguishing between the tokens of these sequences and the abstract sequences.

Both molecular sequences (DNA and RNA molecules as well as proteins) and syntactic sequences (strings like "*ACAC*" and representations in biological databases) have the same number of parts which directly depends on the length of the sequence (i.e., the number of sequence atoms). A molecule or sequence of length 4 will always have 10 parts: itself as a reflexive part, 2 parts of length 3, 3 parts of length 2 and 4 parts of length 1. Abstract sequences, however, are based on equivalence classes (with respect to the relation ≡) of sequence tokens. If multiple parts of a sequence representation represent the same abstract sequence, they are only counted once. Therefore, *ACAC *has the abstract sequences represented by *ACAC*, *ACA*, *CAC*, *AC*, *CA*, *A *and *C *as part, while *CAAC *has the abstract sequences represented by *CAAC*, *CAA*, *AAC*, *CA*, *AA*, *AC*, *A *and *C *as part.

### Implementation and evaluation

We implemented the axiom system using the SPASS first-order theorem prover [12]. The implementation can be found on our project webpage [4]. Due to the restriction of SPASS to first-order logic, we could not implement the axiom 28 requiring connectedness of sequences as well as the condition that ≡ is the minimal relation satisfying axiom 35. These axioms necessitate the use of second-order logics and their implementation would require a theorem prover for higher-order logics.

We employed the SPASS theorem prover on our axioms and attempted to prove the proposition *ϕ *⋀ ¬*ϕ*. If this logical contradiction can be derived from the axioms we provide, our axioms would be inconsistent.

On the other hand, if our axioms are consistent, we expect SPASS to never terminate, because, in the general case, an automated consistency proof for first-order theories is impossible [13].

The SPASS theorem prover could not find a proof for the contradictory statement *ϕ *⋀ ¬*ϕ *in three weeks time on an Intel^® ^Xeon^® ^with 2.5 GHz and 32 GB of memory. However, this is merely an indication for consistency. A formal proof of the consistency, e.g., through the construction of a model, is subject to future work.

Additionally, mere consistency is no indicator for the applicability of the axiom system, or how well it describes the underlying biological reality. In particular, the theory could be consistent yet permit unwanted inferences. We tested the axioms with some basic inferences, i.e. the existence of a sequence, a token, a junction, two non-identical sequences, etc., without detecting an inconsistency.

## Discussion

### Three levels of distinction

A corollary from this ontology of sequences is the necessity to distinguish between the abstract sequences and their tokens. Abstract sequences are abstract entities, independent from space and time, and they can have *tokens*, i.e., physical manifestations that exhibit the structure specified by the sequences. Abstract sequences are similar to universals or ontological categories. Tokens are physical entities that are located in space and time.

The major difference between abstract sequences and their tokens are their identity conditions and the resulting mereology. While there is only one abstact sequence "A", there can be many tokens of that sequence. The tokens can be distinguished in two kinds: syntactic sequences and molecular sequences. The latter are molecules that exhibit the sequential structure determined by the abstract sequence, while the first are syntactic representations of sequences of molecules. The tokens are material objects that are identified, among others, by their position in space and time. Therefore, while the abstract sequence "AA" has only the abstract sequence "A" as proper part, there will always be two tokens of "A" as part of a token of the abstract sequence "AA". As a result, the number of parts of abstract sequences is, in general, not the same as the number of parts of the sequences' tokens.

### Applications of the axiom system

The consequences that can be drawn from the axioms leads to important applications in the development and design of biomedical ontologies. An important consequence is the need for multiple **part-of **relations. Depending on the domain of application, **part-of **has different properties: different axioms hold in different domains.

In our investigation, the largest difference holds between the tokens (molecular or syntactic sequences) and the abstract sequences. While we employed an atomar extensional mereology for the tokens, the **aPO **relation between abstract sequences does satisfy atomicity, but neither the strong nor the weak supplementation principles. The weak supplementation principle is a consequence of the strong supplementation principle (see 17) and states that, if *x *is a proper part of *y*, then there must be some part of *y *which is disjoint from *x*. For abstract sequences, this would be:(39)

However, this axiom does not hold for abstract sequences. This is due to the fact that the **aPO **relation is based on equivalence classes (via ≡) of tokens. For example, the abstract sequence represented by *A *is a proper part (**aPO**) of the abstract sequence represented by *AA*, yet there is no part of *AA *that is disjoint from *A*.

Although our axioms are specific for biological sequences, a parallel can be drawn to other kinds of information objects, such as those covered by the information artifact ontology [14]. The **part-of **relation for information entities, or, more generally, generically dependent continuants (see section) is fundamentally different from the **part-of **relation between material objects.

This observation shows that axiom systems such as the one we provide for biological sequences help to facilitate interoperability between ontologies. They permit the detection of inconsistencies and help to distinguish between different categories and relations based on the properties these categories and relations have.

### Representing hypothetical and faulty sequences

Any ontology of sequences must permit the representation of hypothetical sequences, i.e., sequences that are not the sequences of a molecule. These sequences play a major role in molecular biology. Syntactic sequences that are obtained using current sequencing technologies will often contain errors (i.e., do not exactly correspond to the molecular sequence). Therefore, any sequence of sufficient length that is obtained through these sequencing techniques, such as the sequence of the human chromosome 20, will contain *errors *and there may not be any molecule that exhibits the structure specified by the sequence. Furthermore, randomized sequences are generated and used in bioinformatics analyses and the modeling of evolutionary processes. Whenever these sequences have sufficient length, they will likely represent no molecule.

In an ontology of sequences, it is therefore important to represent syntactic sequences independently from molecules. Syntactic sequences convey information about molecules only *if *there are molecules with the given structure. For an understanding of biological sequences and modelling of the information they convey, all three levels are necessary.

However, some ontologies explicitly exclude abstract entities. One possibility in these ontologies is to model abstract sequences as dependent entities, which depend on certain physical objects. In this case, care must be taken to select the physical entities on which sequences depend; a hypothetical or faulty sequence which has no molecules as tokens cannot be existentially dependent on molecules with the structure specified by the sequence. The sequence specifies the structure of a molecule only if the sequence has molecular tokens.

### Use of first and higher order logic

The axiom system that we developed for sequences is based on second order logic. Satisfiability of a formula is not decidable in second order logic. On the other hand, logics for which satisfiability is decidable such as propositional logic or certain description logics are not sufficiently expressive for our purpose. In particular, connectedness of a sequence is a second order notion; no axiom in first order logic can completely capture the notion of connectedness. Therefore, an expressive logic is necessary to formulate crucial properties of sequences.

While no sound and complete automated reasoner exists for second order logic, theorem provers such a SPASS [12] can be used to assist a user in the inference of theorems from the provided axioms. The alternative to the use of an expressive logic to represent the axioms of the ontology is to restrict the axiom system to a weaker, decidable logic such as the description logic implemented in OWL. However, essential features of the domain would have to be omitted in this case.

It is currently a property of most biomedical ontologies that they use a weak, decidable logic such as the logic defined by OWL, and add natural language definitions to the specified classes and properties to provide their intended meaning. This yields a large part of the ontology that remains informal and therefore ambiguous.

On the other hand, an expressive axiom system that captures large parts of the domain can be used to develop weaker representations for specific purposes. We have only implemented the first order fragment of our axiom system in the SPASS theorem prover. Similarily, it is possible to construct theories in OWL that are compatible with our axiom system. In addition, based on the axiom system we provide, compliant database schemata, software models or other conceptual representations can be constructed. Using natural language for the definition of ontological categories does not permit such a reuse in a consistent manner due to the lack of a formal semantics for natural language.

### Future work

The axiom system we provide is in its first version and has changed substantially during development. We intend to continue development in close collaboration with ontology developers to both increase the usability of the axiom system and improve its clarity. In particular, we plan to carefully examine the second-order axioms to identify potential first-order axioms or axiom schemata that can be used instead of the second-order axioms. Furthermore, the axiom system we present is not complete, and further axioms can be added to increase the strength of the axiomatization.

Additionally, we are investigating possibilities for automatically selecting axioms that can be expressed in a weaker subset of predicate logics than used here. While it is trivial to project the axiom system to first order logics by omitting the two second order axioms we described, axioms in an even weaker logics than first order logics are useful. In particular, for an application within the Semantic Web, we plan to identify a large subset of axioms that can be formulated in the description logic , which forms the basis of the Web Ontology Language (OWL) 2.0. Furthermore, for the application of the axioms within the OBO, a translation to the logic specified by the OBO Flatfile Format [15] could be developed.

## Conclusion

We provide an axiom system for sequences in predicate logics. Most of the axioms are available in first-order logics, although some require the use of second-order logics. The axiom system is intended to serve as a foundation of the Sequence Ontology's top-level categories *Sequence *and *Junction*. As a corollary from the axiom system we developed, we introduced two categories of sequence tokens, which we called *Syntactic sequence *and *Molecular sequence*, and the category *Abstract sequence*. We find that in order to understand *sequence*, it is necessary to consider the tokens of sequences.

The axiom system we provide is not based on a particular top-level ontology, but is compatible with multiple top-level ontologies. We discuss how to include the theory of sequences in the BFO, DOLCE and GFO top-level ontologies. Depending on the top-level ontology used, sequences and junctions are considered different kinds of entities: from generically dependent continuants over abstract individuals to higher-order categories.

This axiom system for sequences is - to the best of our knowledge - the first extensive axiom system for basic categories of an OBO Foundry ontology. With increasing demands for semantic interoperability and information flow between OBO and OBO Foundry ontologies, the importance of developing axiom systems likely will increase, because only axioms can provide a formal specification of a category's meaning, and therefore provide the foundation for automated inferences, information flow and integration. The new axioms are implemented for the SPASS theorem prover and are freely available from our website [4].

## Authors' contributions

RH conceived of the study; HH, RH and JK discussed and designed the basic ontological framework; HH and RH formalized the ontological framework; RH drafted the manuscript and implemented the axioms; HH and JK supervised the project. All authors read and approved the final manuscript.
